# Sustained high blood pressure and 24-h ambulatory blood pressure monitoring in Tanzanian adolescents

**DOI:** 10.1038/s41598-021-87996-0

**Published:** 2021-04-16

**Authors:** Mussa K. Nsanya, Philip Ayieko, Ramadhan Hashim, Ezekiel Mgema, Daniel Fitzgerald, Saidi Kapiga, Robert N. Peck

**Affiliations:** 1 Mwanza Intervention Trials Unit/National Institute for Medical Research, Mwanza, Tanzania; 2 London School of Hygiene and Tropical Medicine, London, UK; 3 Weill Cornell Medical College, New York, USA

**Keywords:** Paediatric research, Epidemiology

## Abstract

Estimates for prevalence of high blood pressure (BP) among adolescents in Africa vary widely and few studies, if any, have documented the results of the recommended stepwise BP screening. In this cross-sectional study in Tanzania, we aimed to estimate prevalence of sustained high BP in 3 public secondary schools using the American Academy of Pediatrics BP screening strategy. On Day 1, one screening automated office BP (AOBP) measurement (Step 1) was followed by two more AOBP measurements (Step 2). Repeat AOBP measurements were obtained after about one month on adolescents with high AOBP measurements on Day 1 (Step 3). Participants with sustained high BP underwent 24-h ambulatory BP monitoring (step 4). Of all 500 enrolled participants, the prevalence of high blood pressure at each step in the process was 36.6% (183), 25.6% (128), 10.2% (51), and 2.6%(13) respectively for Steps 1–4. All except 6 students completed all 4 steps of the BP screening algorithm as indicated. We conclude that diagnosis of hypertension in African adolescents should use multiple AOBP measurements over multiple days followed by 24-h ABPM. Screening for high BP in school settings appears to be feasible and could provide a platform for cardiovascular disease education and health promotion.

## Introduction

High blood pressure (BP) and cardiovascular diseases (CVDs) are emerging in epidemic proportions among adolescents and young adults in sub-Saharan Africa (SSA). In a population survey conducted in Tanzania and Uganda, the prevalence of high BP among young people ranged from 3 to 19% with males having higher estimates than females^1^. In SSA, the rising prevalence of high BP has a disproportional impact on individuals, families, health systems and economies due to relatively young age when the CVD related deaths occur^2,3^.

Also, there are gender differences in distribution of risk factors to CVDs. A population survey among young adults living in urban setting in Tanzania found that compared to men, women have 4.3 higher odds of obesity and 3-folds greater odds for metabolic syndrome. In contrast, women had 50% lower odds for hypertension^4^. These differences have a physiological origin and may track back to childhood and adolescences^5^. However, in SSA cultural norms which define patterns of diet and physical activity may partly add to these differences^6^.

In addition, primary health care facilities in SSA are poorly prepared for diagnosis and management of high BP^7–9^ leaving few adolescents and young people screened for high BP. This situation sets back efforts for cardiovascular health promotion in this population.

However, prevalence estimates for high BP among adolescents and young people in SSA varies widely across studies^10–16^. This is partly due to differences in sampling methods and BP measurement procedures and failure to implement the stepwise approach that is recommended in clinical guidelines^10,17^. To the best of our knowledge, reported studies from Africa have yet employed the BP screening strategy recommended by the 2017 American Academy of Pediatrics (AAP) guideline. In settings where childhood malnutrition is common, potential errors could arise from BP misclassifications in stunted subjects^18^. Additionally, automated office BP (AOBP) has remained the mainstay of BP measurement^15^ despite recommendation on use of 24-h ambulatory BP monitoring (24-h ABPM) as a reliable and accurate tool for confirming high BP which is more predictive of future cardiovascular disease^19,20^ .There is scarcity of data on use of 24-h ABPM among adolescents in sub-Saharan Africa^21,22^, raising concerns on the prevalence estimates for high BP among adolescents.

We therefore conducted a cross sectional study among 500 secondary school adolescents in SSA. We screened for high BP using stepwise procedure recommended by the 2017 American Academy of Pediatrics (AAP) hypertension guidelines^18^. Our overall objective was to determine the outcome of employing the 2017 American Academy of Pediatrics hypertension screening guideline among adolescents in school settings of Tanzania. Our specific objectives were: (1) to determine prevalence and correlates of sustained high BP (2) to describe results of ABPM performed on adolescents with sustained high BP and (3) to describe how CVD risk profiles of these adolescents differ by gender.

## Results

### Enrollment summary

Of the 952 randomly selected participants in the three schools (1st school—350, 2nd school—302 and 3rd school—300), 545 (57.3%) obtained parents/guardians informed written consent for participation. And of those with parental consent, 500 (91.7%) gave their written informed assent and were enrolled in the study. Reasons for not being enrolled among those with parental consent were; declining assent 41 (7.5%) and being considered ineligible 4 (0.08%) due to short duration (less than 1 year) of residence in Mwanza city. There were no systematic differences in age, sex and level of education between participants who were enrolled from those who declined to participate. AOBP measurements at day 1 were available for all 500 enrolled participants and 128 participants who were eligible for repeat BP measurements at day 2.

Of all 500 enrolled participants, 51(10.2%) had sustained high BP and were eligible for 24-h ABPM but we successful conducted the study in 45 (88.2%). Reasons for not performing 24-h ABPM were: death (1), refusal (1) and moving out of Mwanza city (4). One 24-h ABPM measurement needed to be repeated due to insufficient data quality. In the end, ABPM data from all 45 participants were available for analysis.

### Background characteristics

In Table 1, we present the overall background characteristics of the 500 enrolled study participants by their gender groups. The mean age was 13.9 (0.8) years. Majority of the participants were females (56.6%); had lived in Mwanza city for more than 10 years (85.2%); were in lower wealth index (57.6%) and had their BP being measured for the first time in this study (91.2%).

**Table 1:** Background characteristics of adolescents enrolled in a cross sectional study in Mwanza City, Tanzania (N = 500).

Characteristic	Male and female N = 500 n (%)	Male N = 217 n (%)	Female N = 283 n (%)	*P* value
**Socio-demographic and economic factors**				
Age (years)^1^	13.9 (0.8)	14.1 (0.8)	13.8 (0.8)	0.0010
Duration of residence in Mwanza City				0.0020*
More than 10 years	426 (85.2)	197 (90.8)	229 (80.9)	
Wealth index group^2^				0.20*
Lower	288 (57.6)	132 (60.8)	156 (55.1)	
Middle/upper	212 (42.4)	85 (39.2)	127 (44.9)	
Measured BP for the first time	456 (91.2)	197 (90.8)	259 (91.5)	0.77
**Behavior factors**				
Ever smoked tobacco	5 (1.0)	3 (1.3)	2 (0.7)	0.45
Ever drunk alcohol	46 (9.2)	31 (14.3)	15 (5.3)	0.0010
Fruits servings consumed per day				
0	311 (62.2)	137 (63.1)	174 (61.5)	
1	111 (22.2)	38 (17.5)	73 (25.8)	0.062*
2–4	61 (12.2)	33 (15.2)	28 (9.9)	
≥ 5	17 (3.4)	9 (4.2)	8 (2.8)	
Vegetables servings consumed per day				
0	398 (79.9)	179 (82.5)	219 (77.4)	
1	87 (17.4)	32 (14.8)	55 (19.4)	0.36*
≥ 2	15 (3.0)	6 (2.8)	9 (3.2)	
Teaspoons of sugar per day				
< 10	203 (40.6)	90 (41.5)	113 (39.9)	0.73*
≥ 10	297 (59.4)	127 (58.5)	170 (60.1)	
Minutes spent in moderate physical activity per day				0.55*
≥ 60	194 (38.8)	81 (37.4)	113 (39.9)	
Days spent in vigorous physical activity per week				< 0.0001*
≥ 3	133 (26.6)	25 (11.5)	108 (38.2)	
**Biological factors**				
Body Mass Index (BMI)				
Underweight	57 (11.5)	40 (18.4)	17 (6.0)	
Normal	405 (80.8)	170 (78.3)	235 (83.0)	< 0.0001*
Overweight/obesity	38 (7.7)	7 (3.2)	31 (11.0)	
Pulse rate (beats/min)				
< 60	25 (5.0)	23 (10.6)	2 (0.7)	
≥ 60 to < 90	364 (72.8)	167 (77.0)	197 (69.6)	< 0.0001*
≥ 90	111 (22.2)	27 (12.4)	84 (29.7)	
Stunted growth^3^	70 (14.0)	49 (22.6)	21 (7.4)	< 0.0001
eGFR ^4^				
Normal (≥ 60 ml/min/1.73 m^2^)	448 (89.6)	192 (88.5)	256 (90.5)	0.42*
Renal dysfunction (< 60 ml/min/1.73 m^2^)	52 (10.4)	25 (11.5)	27 (9.5)	

^1^Mean (SD).

^2^Wealth Index: an aggregate variable generated by combining data on parents/guardian’s vital status, occupation and ownership of common household items.

^3^Stunted growth defined according to WHO—height for age below -2 standard deviation (SD).

^4^ Estimated glomerular filtration rate (calculated using the modified Schwartz equation).

P value: compares males and females.

*p value for 2 or more categories.

Of all participants, 62.2% and 79.9% never consumed fruits and vegetables respectively in a typical week. Similar proportion of participants reported less than the recommended levels of moderate and vigorous physical activity respectively. Of all study participants, 59% reported using an equivalent of 10 or more teaspoons of sugar in a typical day. Majority of the participants (80.8%) had normal BMI, however males formed majority (70%) of underweight participants whereas females formed majority (80%) of overweight or obese participants. 14% of all participants were stunted and majority of them (70%) were males. In addition, alcohol drinking (p = 0.0006), vigorous physical activity (p < 0.0001) and resting pulse rate ≥ 90 beats/min (p < 0.0001) showed significant statistical difference between males and females.

52 participants (10.4%) had kidney dysfunction (eGFR ≤ 60    ml/min/1.73 m^2^). Of those 52 participants, 27 (5.4%) had sustained kidney dysfunction on repeating the test six months later. Of all participants, overt proteinuria was present in 6.6% and only 1% and 17.6% had sickle cell disease and trait respectively.

Of 51 eligible participants, 45 underwent 24-h ABPM study and they had a mean age of 14.0 (0.8) years, 56% were females and majority (71%) were in lower wealth index. Additionally, 93% had normal BMI for their age, 11% were stunted and 16% had kidney dysfunction (eGFR below 60 ml/min/1.73 m^2^).

### Prevalence of high AOBP and 24-h ABPM

**Figure 1:**
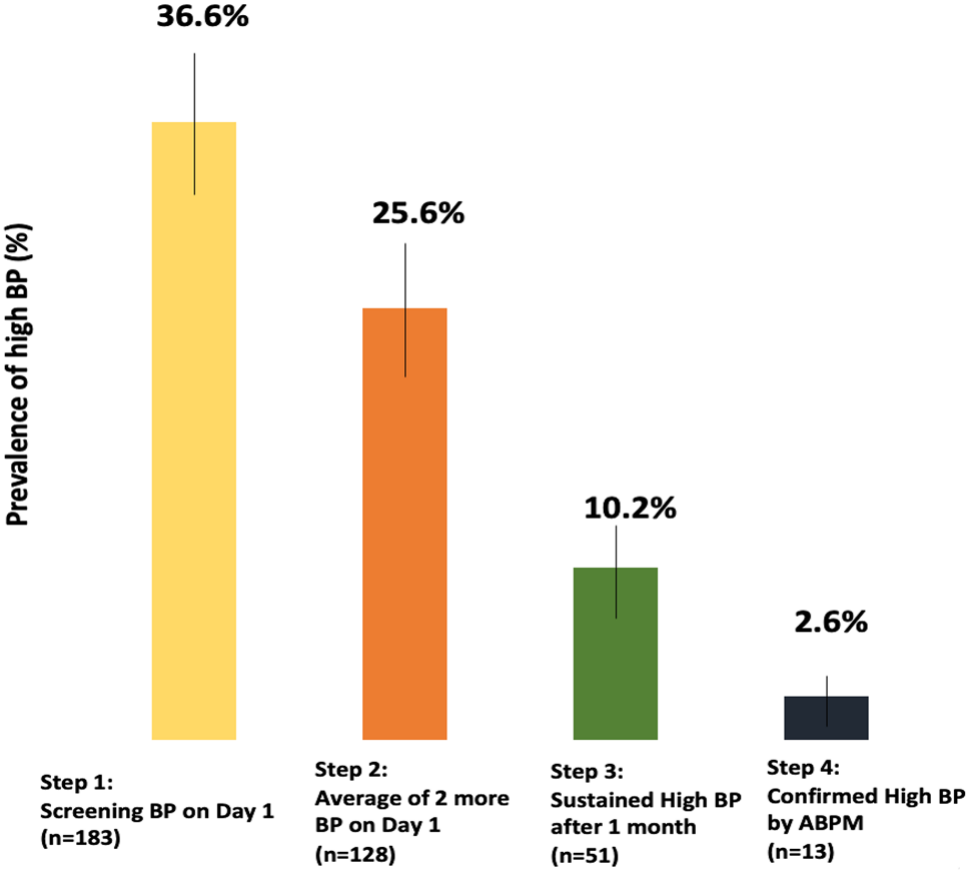
Prevalence estimates of high BP with their corresponding 95% confidence intervals in Tanzanian adolescents according to the stepwise blood pressure screening protocol recommended by the American Academy of Pediatrics (N = 500).

In Fig. 1 , we present the prevalence of high BP in each step of screening using the 2017 American Academy of Pediatrics guideline^18^. In the *first step*, based on the first AOBP measurement obtained on Day 1, the prevalence of high BP was 36.6% (95% CI: 32.5–40.9%). In the *second step*, based on the average of second and third AOBP obtained on Day 1, the prevalence of high BP was 25.6% (95% CI 21.8–29.7%). At the *third step*, based on the average of second and third AOBP repeat measurement obtained about 1 month after enrollment among participants with high BP on Day 1, the prevalence of sustained high BP was 10.2% (95% CI: 7.7–13.2%).

Of the 45 participants with 24-h ABPM studies, 13 (28.9%) had confirmed high BP, of whom 12 had pre-hypertension and 1 had hypertension. The remaining 32 (71.1%) had white coat hypertension. The overall estimate of confirmed high BP using 24-h ABPM during enrollment was therefore (13/500), 2.6% (95%CI, 1.4–4.4%).

In Table 2, we compared socio-demographic, behavior and biological characteristics of participants with normal AOBP (n = 449) to those with sustained high BP (n = 51). In a logistic regression analyses adjusted for age and sex (a priori confounders), smoking cigarette aOR (95%CI)—6.47 (1.04 to 40.4); consuming more than 10 teaspoons of sugar in a typical day aOR (95%CI)—1.92 (1.01 to 3.65) and having resting pulse ≥ 90 beats per minute aOR (95%CI)—1.98 (1.02 to 3.86) were significantly associated with sustained high BP in the multivariable model.

**Table 2:** Comparison of background characteristics of adolescents with sustained high BP (n = 51) to those with normal BP (n = 449) enrolled to a cross-sectional study.

Characteristic	Sustained high BP (n = 51), n (%)	Normal BP (n = 449), n (%)	P value^¥^	Adjusted OR (95%CI)^£^
**Demographic factors**				
^1^Age (years)	14.0 (0.8)	13.9 (0.8)	0.25	1.12 (0.78 to 1.60)
Sex (female)	27 (52.9)	256 (57.0)	0.58	0.87 (0.48 to 1.56)
Duration of residence in Mwanza city (≥ 10 years)	48 (94.1)	378 (84.2)	0.06	
^2^Wealth index class				
Lower	35 (68.6)	253 (56.4)	0.09*	
Middle/upper	16 (31.4)	196 (43.7)		
Measured BP for the first time	48 (94.1)	408 (90.9)	0.44	
**Behavior factors**				
Ever smoked tobacco	2 (3.9)	3 (0.7)	0.03	6.47 (1.04 to 40.4)
Ever drank alcohol	7 (13.7)	39 (8.7)	0.24	
Fruits servings consumed per day				
0	33 (64.7)	278 (61.9)		
1	10 (19.6)	101 (22.5)	0.88*	
2–4	7 (13.7)	54 (12.0)		
≥ 5	1 (2.0)	16 (3.6)		
Vegetable serving per day				
0	39 (76.5)	359 (80.0)		
1	9 (17.7)	78 (17.4)	0.44*	
≥ 2	3 (5.9)	12 (2.7)		
Teaspoons of sugar per day				
< 10	14 (27.5)	189 (42.1)	0.04*	Reference
≥ 10	37 (72.6)	260 (57.9)		1.92 (1.01 to 3.65)
Minutes spent in moderate physical activity per day				
< 60	30 (58.8)	276 (61.5)		
≥ 60	21 (17.7)	173 (38.5))	0.13*	
Days spent in vigorous physical activity per week				
< 3 days	42 (82.3)	325 (72.4)	0.13*	
≥ 3 days	9 (17.7)	124 (27.6)		
**Biological factors**				
BMI categories				
Underweight	2 (3.9)	55 (12.3)		0.25 (0.06 to 1.08)
Normal	48 (94.1)	357 (79.5)	0.04*	Reference
Overweight/Obesity	1 (2.0)	37 (8.2)		0.22 (0.03 to 1.65)
Pulse rate				
< 60	5 (9.8)	20 (4.5)		2.51(0.85 to 7.44)
≥ 60 to < 90	30 (58.8)	334 (74.4)	0.04*	Reference
≥ 90	16 (31.4)	95 (21.2)		1.98 (1.02 to 3.86)
^3^ Stunted growth	6 (11.8)	64 (14.3)	0.63	
Urine protein test results				
Negative	24 (47.1)	200 (44.5)		
Trace	24 (47.1)	219 (48.8)	0.93*	
(30 to > 300) mg/dL	3 (5.9)	30 (6.7)		
Sickle cell test				
Negative	45 (88.2)	362 (80.6)		
Trait	6 (11.8)	82 (18.3)	0.37*	
Diseased	0 (0.0)	5 (1.1)		
^4^Kidney function (eGFR)				
(> 90 ml/min)	7 (13.7)	93 (20.7)		
(60–90 ml/min)	37 (72.6)	311 (69.3)	0.41*	
(< 60 ml/min)	7 (13.7)	45 (10.0)		

^1^Mean (SD).

^2^Wealth Index: an aggregate variable generated by combining data on parents/guardian’s vital status, occupation and ownership of common household items.

^3^Stunted growth defined according to WHO—height for age below -2 standard deviation (SD).

^4^Estimated Glomerular Filtration Rate (calculated using the modified Schwartz equation).

¥Student’s t-test (continuous variable) and Chi squared test (categorical variable).

*Overall p value.

^**£**^Adjusted for age and sex.

In Supplementary Table, we present gender differences in distribution of ABPM characteristics. Males had significantly higher awake diastolic BP load (p = 0.009). In contrast, females had significantly higher ambulatory heart rate while awake (p = 0.0003) and while asleep (p = 0.0006).

## Discussion

In this school-based cross-sectional study of 500 Tanzanian adolescents selected randomly from 3 public secondary schools in Mwanza city, Tanzania, we report results from high BP screening using four different steps of blood pressure screening obtained over the course of several months as recommended by the 2017 American Academy of Pediatrics guideline^18^. Although about 25.6% of all study participants had high BP using AOBP measurements obtained on the first day, only 10% had sustained high BP and less than 3% were confirmed to have high BP using ABPM. Overall, smoking cigarette, consuming more than 10 teaspoons of sugar in a typical day and having high (≥ 90) resting pulse were associated with sustained high BP. Also, traditional, endemic and ABPM-related CVD risk factors were common and differed significantly by gender.

In this study we conducted repeat AOBP measurements only in those identified with high BP during initial measurements. This is the recommended BP measurement strategy among adolescents and has a potential of saving time and other resources which would have been used for follow-up and confirmatory measurements on all participants^18,23^. Becton and colleagues reported that more than 90% of adolescents aged 13–18 years participating in the US National Health and Nutrition Examination Survey (NHANES) had their BP classification remaining the same in follow up measurements and only < 3% had an increase in BP classification^23,24^. Moreover, the high BP screening algorithm we used has a negative predictive value of more than 99%^18^.

Adolescents with sustained high BP were more likely to smoke cigarette, consume more than 10 teaspoonful of sugar and have high resting pulse (≥ 90 beats per minute) (Table 2). These are well established traditional risk factors to high BP which deserve public health intervention. However, it is worth noting that nearly 95% of participants with sustained high BP had normal BMI which is similar to another study in South Africa^25^. This is a different pattern to that reported in developed countries such as USA, where overweight and obesity are the major drivers of high BP among adolescents^18^. Therefore this finding underscores the need to screen BP on all adolescents regardless of their adiposity and to confirm high BP using 24-h ABPM, as recommended by standard guidelines^18,20^. Encouragingly, 90% (45/51) of eligible adolescents and their parents agreed to undertake ABPM test indicating that it is also feasible to conduct 24-h ABPM in Tanzania and it may even be possible to do this in school-based health programs.

Risk factors for CVD are common among adolescents in Tanzania. For example, 62.2% and 79.9% did not consume any serving of fruits and vegetables respectively in a typical week, which is far lower than the recommended 5 daily servings^26^. Since fruits and vegetables are high in potassium, low consumption could contribute directly to high BP in Africa^27^. In addition, 5.4% of participants had confirmed kidney dysfunction (eGFR of < 60 ml/min). We have previously reported that reduced kidney function is common in both children and young adults in Tanzania^28,29^, and is likely related to an interplay of some of neglected tropical diseases, HIV, environmental exposures in mining communities and childhood under nutrition^30^. Therefore, reduced kidney function could represent an important endemic risk factor for early CVD in Tanzania. Importantly, 25% of adolescents with 24-h ABPM data had evidence of nocturnal non-dipping. Nocturnal non-dipping with or without high BP is an established modifiable risk factor for cardiovascular diseases particularly among people of African origin^31^ and has been associated with stroke and left ventricular hypertrophy^32,33^. Further studies are needed to determine longitudinal trajectory of abnormal ABPM characteristic and their association with CVD in later adolescence and early adulthood.

Also, the distribution of CVD risk factors among adolescents in Tanzania differs significantly by gender. For instance, alcohol use, participation in vigorous physical activity, stunted growth and underweight were more common in males, whereas, females were more likely to be overweight or obese and report the recommended level of moderate and vigorous physical activity. Interestingly, females had relatively higher average pulse rate (which is a marker for lower physical fitness). Similar findings have been reported elsewhere in Africa^6,11,12,16^. Also, 24-h ABPM characteristics including heart rate and diastolic BP load varied significantly by gender with male adolescents generally having higher ABPM parameters than their female counterparts^20^. Similar findings have been previously reported^34–36^. While these differences are partly known to have their basis in hormonal and hemodynamic differences between males and females^5^, social and cultural norms which define gender roles could also be contributing to the observed differences in the Tanzanian context^6,37^.

Our study has strengths and limitations. One major strength of our study was the high follow-up rate from initial to repeat AOBP measurements (obtained after about one month) to 24-h ABPM measurements. On the other hand, these are the limitations of this study; we worked only in 3 schools and the results may not be generalisable although this does not impact on the validity of the findings; lack of repeat AOBP measurements and 24-h ABPM data from all participants limited our assessment of ‘masked hypertension’ in this group of African adolescents. Also, the relatively low response of parents/guardians to provide consent for their children’s participation in the study may have introduced selection bias.

In conclusion, sustained high BP among adolescents in Tanzania is common and is associated with traditional risk factors to CVD. Standard procedure for screening and diagnosis of high BP in African adolescents should include multiple AOBP measurements obtained at multiple days and followed by 24-h ABPM. Screening for high BP in school settings appears to be acceptable and feasible in Tanzania. Cardiovascular health education in Tanzanian schools should address the existing traditional and endemic risk factors and take into account gender differences.

## Methods

### Study design, setting and sampling

Between April and August 2018, we collected data from 500 secondary school students in Mwanza city in Tanzania (East Africa). Mwanza is the second largest city located on the southern shores of Lake Victoria with an estimated population of about 1 million people. About 23% of the population in Tanzania is adolescents and the overall secondary school net enrollment is about 33%^38^ In Tanzania, secondary education generally begins around 13 years of age and lasts for 4 years of ordinary level and 2 more years for advanced level^39^.

For enrollment, we initially prepared a comprehensive list of day secondary schools (20 public and 1 private) which enroll both male and female students and are located within a radius of 5 km from Mwanza city centre. From this list, we randomly selected three public schools to participate in the study.

Then at each school, a de-identified list of students in first and second year of secondary school education with information on their date of birth was obtained. At each school about 300 students aged 11–15 years were selected using random sampling stratified by age to ensure proportional enrollment across the eligible age range.

Parents/guardians of selected students were invited to meet with the study team at the school to obtain information about the study and provide consent for their children to be enrolled in the study. Students whose parents/guardians did not attend the meeting at school were given reminder letters to take home. The letter provided detailed study and contact information and consent forms for the parents/guardians to sign if they wanted their children to join the study. Then we sought assent from students with documented approval from parents. Students who had lived in Mwanza city for less than one year were excluded due to concerns that they may move away from the city before the end of the study period.

### Data collection procedures

A structured questionnaire adapted from the WHO STEPwise approach to non-communicable diseases (NCDs) risk factors survey (STEPS instrument)^40^ was used. The questionnaire was translated into the local language (Kiswahili) and then independently back-translated to English for validation. We obtained information about socio-demographic characteristics and potential risk factors for high BP. We also collected information about parent/guardian’s vital status, occupation and ownership of common household items which were used to generate categories of participant’s wealth index. We collected data using handheld electronic devices (TECNO Android Tablets) programmed to check for accuracy and consistency of entered data.

After the interviews, we conducted physical examination to collect anthropometric measurements. Height was measured in an upright standing posture on bare foot and without head ornament, using a portable stadiometer (SECA 213, SECA GmbH & co. KG., Hamburg, Germany) at a precision of 0.1 cm. Waist and hip circumference were measured over a single layer of clothing using a tape measure (SECA 201, SECA GmbH & co. KG, Hamburg, Germany) at a precision of 0.1 cm. Weight was measured with a participant in an upright standing posture, in light clothing and on bare foot, using a digital weight scale (SECA 876 flat scale, Seca GmbH &co. KG., Hamburg Germany) at a precision of 0.1 kg. The weight and height measurements were used to calculate the body mass index (BMI) which was categorized into underweight, normal weight, overweight or obesity using the WHO’s BMI for age Z-scores^41^.

We obtained AOBP measurements using a validated monitor (OMRON Model BP791IT—HEM-7222-ITZ, Omron Health care Inc., Lake Forest, Illinois, USA) while participants were seated in an upright position with an appropriate cuff size as determined from the participant’s right mid-upper arm circumference (MUAC). The BP measurement procedure was fully explained and done after resting for at least 5 min in a booth, the participant started the BP machine and three consecutive BP measurements and counts for heart beats per minute were automatically obtained at an interval of 1 min and precision of 0.1 mmHg. The first AOBP measurements obtained on ‘Day 1’ was used to screen for high BP (step 1) using the screening algorithm of the 2017 American Academy of Pediatrics (AAP) guideline^18^. Participants aged ≥ 13 years and whose AOBP measurement were ≥ 120/80 mmHg, or those aged < 13 years and whose AOBP measurement was ≥ 90th percentile for their age, height and sex screened positive for high BP. Additional two AOBP measurements were obtained on the same day 1 and their average were compared to reference table for age, sex and height (step 2). If average AOBP measurements were high (≥ 90th percentile for age, sex and height) on day 1, repeat AOBP measurements were obtained after about one month (‘*Day 2′*) using same procedure (step 3). The average of last two AOBP measurements obtained on *Day 2* was used to obtain *final* BP percentiles using BP reference tables which takes into account participant’s age, sex and height [also available in a Statistical Analysis System (SAS) code] in the guideline^18^. Participants with final systolic and/or diastolic AOBP measurement ≥ 90th percentiles for their age, sex and height were regarded as having sustained high BP and were eligible for 24-h ABPM (step 4).

After physical examination and AOBP measurements, we collected 5 mL of venous whole blood for malaria, hemoglobin electrophoresis, and serum creatinine tests. While in the field, we used two drops of blood to test for malaria using rapid diagnostic test (ACCESS BIO, 65 Clyde Road, New Jersey, USA), and five drops of blood for preparing dried blood spot (Whatman 903 filter paper) samples. The remaining blood sample was transported to the central laboratory at the National Institute for Medical Research (NIMR—Mwanza) for further processing and testing. We also collected about 40 mL of urine sample for urine tests including 10 mL which was used for urine strip analysis (ACON laboratories Inc, San Diego, California—USA) at the field. We communicated abnormal results to the respective participants in presence of their parents/guardians. Additionally, participants with sickle cell trait or disease were counseled on what the results meant, what to expect and what they were supposed to do to modify the course of their disease as well as on the importance of screening for the disease before marriage.

### Laboratory procedures

At the central NIMR laboratory, about 30 mL of the remaining urine was centrifuged and aliquoted in two cryotubes (1.8 mL each) and stored at − 80 °C for future testing.

The dried blood spot samples were kept overnight in a clean environment to air-dry. They were then sealed in labeled bags and stored at a temperature of − 80 °C. Then, we performed hemoglobin electrophoresis to characterize participants’ hemoglobin as normal, sickle cell trait or sickle cell disease hemoglobin (MULTIPHOR II Electrophoresis Unit, 751 84, Uppsala Sweden).

The remaining whole venous blood was centrifuged to separate serum and two 1.8 mL aliquots were prepared and stored at − 80 °C for future testing. One of the stored aliquots was used to test for serum creatinine (A25 BIOSYSTEMS SA. Costa Brava, 30. Barcelona, Spain) whose results were used to calculate Estimated Glomerular Filtration Rate (eGFR) based on the modified Schwartz equation^42^.

### 24-h ambulatory blood pressure monitoring (24-h ABPM) data collection procedure

The 24-h ABPM were conducted by a trained study nurse using a validated monitor (OSCAR 2, SunTech Medical) during routine school days^20^. The monitor was programmed using AccuWin Pro 4 software to obtain BP measurements every 15 min for 24 h^20^. Mid-upper arm circumference was used to determine the appropriately sized cuff, which was placed on a non-dominant arm and connected to the battery powered ABPM monitor using rubber tubing. Then we started the monitor and confirmed that it was functioning by observing two consecutive readings before allowing the participant to leave. After 24 h, we removed the ABPM monitor and downloaded the data. Sleep and wake times were defined using participant’s diary as recommended for adolescents^43^. At least 20 and 7 readings during day and night respectively were required for a valid 24-h ABPM test. Height, gender and age were factored in to obtain the participant’s BP category. Times when participants were active or distressed were noted and used to interpret results. Pre-specified 24-h ABPM characteristic were extracted to data collection forms for double data entry using Open Clinica version 3.1.4. Participants with high BP using 24-h ABPM were regarded as having a confirmed diagnosis and counseled on diet and lifestyle changes and their BP were followed up during the study (if they had pre-hypertension) OR referred to specialist care (if they had hypertension)^18^.

### Statistical analysis procedures

We had three AOBP measurements obtained on *Day 1* (for all participants) and three additional follow-up AOBP measurements obtained after about 1 month from those with high BP on *Day 1*. We estimated the prevalence of high BP and their corresponding 95% confidence intervals, at each step in the screening process, using interval estimate for binomial proportion. The *first step*, used the first AOBP measurement (in a series of three) obtained on *Day 1* to screen for high BP using the ‘*screening algorithm’* of the 2017 American standard guideline^18^. Participants with BP ≥ 120/80 mmHg if aged ≥ 13 years or BP ≥ 90th percentile if aged < 13 years were regarded as having high BP. The *second step* used the average of the second and third AOBP measurements (in a series of three) obtained on *Day 1* to screen for participants with high BP using the BP percentile tables. The *third step* only involved participants with high average AOBP on day 1. We used average of second and third follow-up AOBP measurements (in a series of three) obtained on *Day 2* to obtain their ‘*final’* BP using the BP percentile tables^18^. Participants whose systolic and/or diastolic BP ≥ 90th percentiles for their age, sex and height were regarded as having sustained high BP and underwent 24-h ABPM. We then categorized 24-h average BP as either normal BP OR pre-hypertension OR hypertension OR severe hypertension, depending on ABPM 95th percentile score and/or systolic or diastolic BP load ≥ 25% as recommended by the 2014 American Heart Association guideline^20^. Participants whose average 24-h BP was categorized as pre-hypertension, hypertension or severe hypertension were regarded as having confirmed high BP while those with normal BP using 24-h ABPM were regarded as having white coat hypertension.

Participants’ demographic, biological and behavioral characteristics were separately summarized for males and females using median [IQR] for continuous variables and proportions for categorical variables. To test for statistical significant differences between males and females, Student’s T-test (continuous variable) and Chi-squared test (for categorical variable) were used.

To assess for factors associated with sustained high BP, we compared socio-demographic, behavior and biological characteristics of participants with sustained high BP to those with normal AOBP measurements. Student’s T test (continuous variable) and Chi-squared test (categorical variable) were used to test for statistical significant differences between the groups. Logistic regression analyses adjusted for age and sex (priori confounders) were conducted on characteristics with statistical significance (p ≤ 0.05) during initial analyses.

Student’s T test (continuous variables) and (Fisher’s exact test) were used to assess for significant differences in ABPM characteristics between males and females. In order to adjust for potential type 1 error resulting from multiple comparisons, we only reported ABPM characteristics with p-value < 0.01 as statistically significant. Statistical analysis was performed using STATA IC version 14 (StataCorp, College Station, Texas, USA).

### Ethical considerations

The study was approved by the ethics committee of the Tanzania National Institute for Medical Research (NIMR/HQ/R.8a/Vol.IX/2452) and the Weill Cornell Medical College Institutional Review Board (#1612017830). Informed written consent from parents or guardians was obtained before participants were approached for their informed written assent. To ensure confidentiality, unique study identity numbers were used. All methods were performed in accordance with the relevant guidelines and regulations.

## Supplementary Information

Supplementary Information 1.
