# Supplementary material for: Sustained high blood pressure and 24-h ambulatory blood pressure monitoring in Tanzanian adolescents
Source: Sci Rep. 2021 Apr 16;11:8397. doi: 10.1038/s41598-021-87996-0 (PMC8052360; doi:10.1038/s41598-021-87996-0)
Supplement: Supplementary file 1 — Supplementary Information 1. [file 41598_2021_87996_MOESM1_ESM.pdf]

Sustained High Blood Pressure and 24-Hour Ambulatory Blood Pressure Monitoring in Tanzanian Adolescents

Mussa K. Nsanya [1*], Philip Ayieko [1,2], Ramadhan Hashim [1], Ezekiel Mgema [1], Daniel Fitzgerald [3], Saidi Kapiga[1,2], Robert N. Peck [1,3]

[1] Mwanza Intervention Trials Unit / National Institute for Medical Research, Mwanza, Tanzania

[2] London School of Hygiene and Tropical Medicine, London, UK

[3] Weill Cornell Medical College, New York, USA

[*]**Corresponding Author**: Correspondence concerning, and reprints request of this article should be addressed to Mussa K. Nsanya, e-mail: mussa.nsanya@mitu.or.tz.

Supplementary Table: Distribution of ambulatory BP characteristic by gender among secondary school adolescents with sustained hypertension in Mwanza City, Tanzania (N=45).

Ambulatory BP Characteristic	**Males and Females (N=45)** N (%) or Mean (SD)	**Males (N=20)** N (%) or Mean (SD)	**Females (N=25)** N (%) or Mean (SD)	**¥ P value**
Confirmed high BP (24 hour ABPM)	13 (29.0%)	5 (25.0%)	8 (32.0%)	NS
Average office systolic BP (mmHg)	125.9 (7.4)	127.4 (7.5)	124.7 (7.2)	NS
Average office diastolic BP (mmHg)	76.8 (6.7)	76.2 (6.1)	77.3 (7.3)	NS
Average office heart rate (bpm)	80.9 (16.9)	74.0 (13.0)	86.4 (17.9)	0.01
24-hour systolic ABPM (mmHg)	111.6 (6.7)	113.7 (8.2)	110.0 (4.7)	NS
24-hour diastolic ABPM (mmHg)	64.8 (4.5)	64.8 (5.2)	64.9 (3.8)	NS
Asleep systolic ABPM (mmHg)	102.8 (7.4)	104.9 (9.2)	101.2 (5.3)	NS
Asleep diastolic ABPM (mmHg)	55.2 (4.8)	54.9 (5.6)	55.4 (4.1)	NS
Awake systolic ABPM (mmHg)	117.3 (6.8)	119.5 (7.9)	115.6 (5.4)	NS
Awake diastolic ABPM (mmHg)	70.6 (5.9)	70.4 (7.3)	70.8 (4.5)	NS
Awake ABPM heart rate (bpm)	84.6 (12.3)	78.0 (11.0)	90.0 (10.8)	0.001
Asleep ABPM heart rate (bpm)	68.3 (10.7)	62.8 (10.6)	72.7 (8.6)	0.001
Heart rate non-dipper (< 10%)	2 (4.4)	1 (5.0)	1 (4.0)	0.01
Systolic BP dip (%)	12.4 (4.0)	12.3 (4.6)	12.5 (3.6)	NS
Diastolic BP dip (%)	22.3 (5.7)	23.2 (6.3)	21.5 (5.2)	NS
Systolic BP non-dipper (<10%)	11 (24.4%)	5 (25.0%)	6 (24.0%)	NS
Diastolic BP non-dipper (<10%)	1 (2.2%)	0 (0.0%)	1 (4.0%)	NS
Combined BP non-dipper (<10%)	11 (24.4%)	5 (25.0%)	6 (24.0%)	NS
24-hour systolic BP load (%)	12.5 (11.0)	13.5 (13.5)	11.7 (8.6)	NS
24-hour diastolic BP load (%)	13.3 (10.2)	16.6 (12.9)	10.7 (6.5)	NS
Awake systolic BP load (%)	14.6 (11.0)	14.7 (12.5)	14.5 (9.8)	NS
Awake diastolic BP load (%)	15.8 (11.9)	20.4 (14.7)	12.1 (7.6)	0.01
Asleep systolic BP load (%)	9.5 (14.0)	12.1 (18.5)	7.4 (8.8)	NS
Asleep diastolic BP load (%)	9.7 (11.3)	11.7 (15.0)	8.2 (7.2)	NS
Morning BP surge (mmHg)	21.3 (13.2)	20.6 (12.2)	21.8 (14.1)	NS
Ambulatory Arterial Stiffness Index (AASI)	0.25 (0.12)	0.24 (0.13)	0.26 (0.12)	NS

¥ Student's t-test (continuous variable) and Fisher's exact test (categorical variable)
NS – Not statistically significant ($p > 0.01$)
